# Supplementary material for: Deciphering the mechanism of glutaredoxin-catalyzed roGFP2 redox sensing reveals a ternary complex with glutathione for protein disulfide reduction
Source: Nat Commun. 2024 Feb 26;15:1733. doi: 10.1038/s41467-024-45808-9 (PMC10897161; doi:10.1038/s41467-024-45808-9)
Supplement: Supplementary file 1 — Supplementary Information [file 41467_2024_45808_MOESM1_ESM.pdf]

# Supplementary Information

## Deciphering the mechanism of glutaredoxin-catalyzed roGFP2 redox sensing reveals a ternary complex with glutathione for protein disulfide reduction

**Fabian Geissel[1a], Lukas Lang[1a], Britta Husemann[1], Bruce Morgan[2] & Marcel Deponte[1*]**

[1]Faculty of Chemistry, Comparative Biochemistry, RPTU Kaiserslautern, D-67663 Kaiserslautern, Germany

[2]Institute of Biochemistry, Centre for Human and Molecular Biology (ZHMB), Saarland University, D-66123 Saarbrücken, Germany

*Correspondence and requests for materials should be addressed to M.D. (deponte@chemie.uni-kl.de)

[a]These authors contributed equally

## Supplementary Figure 1

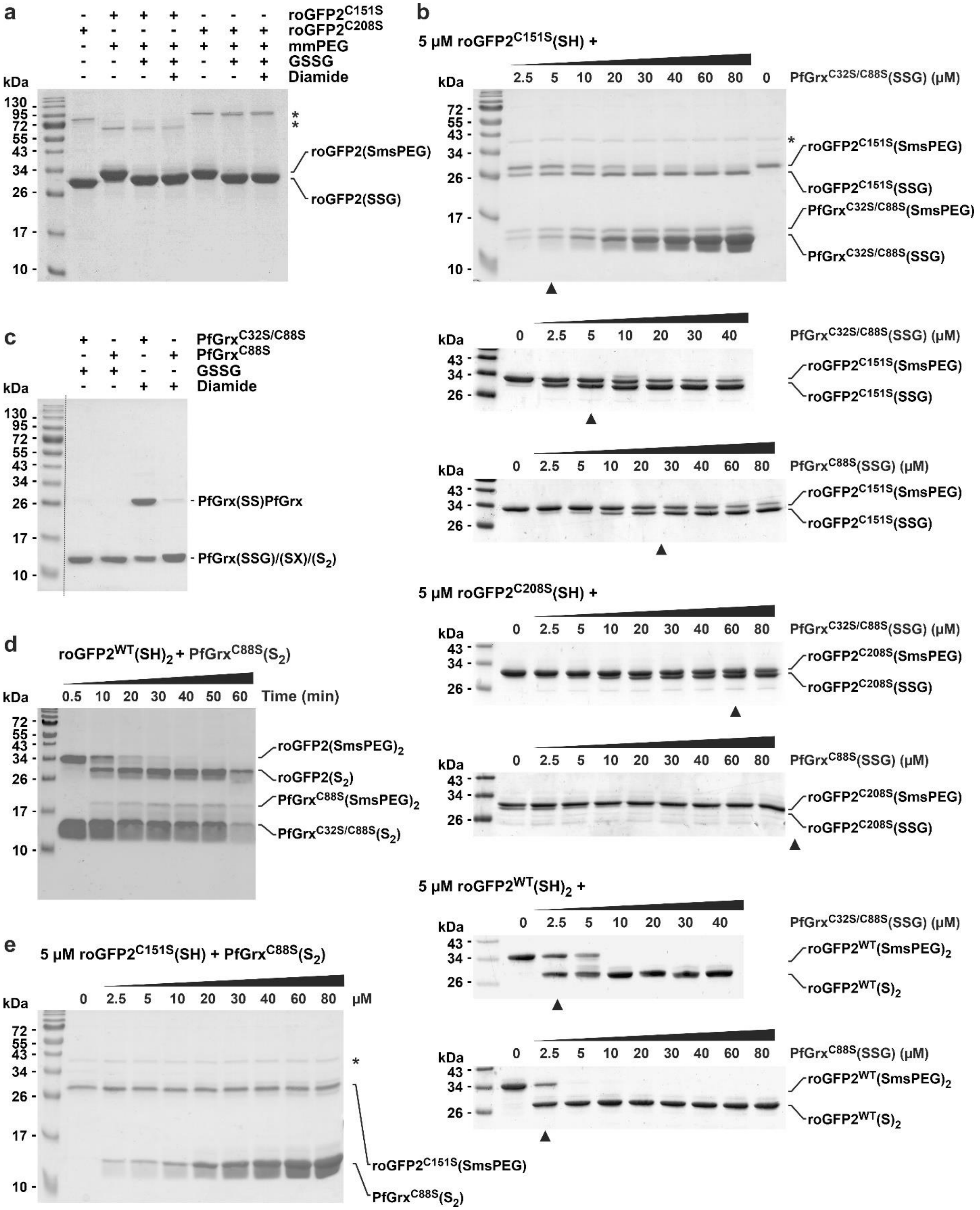

**Supplementary Figure 1 | Electrophoretic mobility shift assays.** (**a**) Successful glutathionylation of roGFP2$^{C151S}$(SH) and roGFP2$^{C208S}$(SH) yielding roGFP2$^{C151S}$(SSG) and roGFP2$^{C208S}$(SSG) was confirmed by alkylation protection assays and non-reducing SDS-PAGE. Samples that were pre-treated with GSSG revealed no altered electrophoretic mobility following the addition of the alkylating agent mmPEG$_{24}$ in contrast to samples without GSSG. Untreated roGFP2$^{C208S}$(SH) served as a control. The addition of Diamide did not result in the formation of disulfide-bridged dimeric roGFP2. The upper bands that are labelled with asterisks were therefore minor impurities. (**b**) The relative redox potentials of the indicated PfGrx and roGFP2 redox couples were estimated by mixing 5 µM of the roGFP2 variant with the indicated concentrations of glutathionylated PfGrx followed by alkylation of thiol groups with mmPEG24 and subsequent non-reducing SDS-PAGE. The equilibration between 5 µM roGFP2$^{C151}$(SH) and 5 µM PfGrx$^{C32S/C88S}$(SSG) shown on top served as a reference and resulted in even band intensities for the roGFP2 and PfGrx$^{C32S/C88S}$ redox couples in accordance with a highly similar redox potential (see arrowhead). Comparative analyses of six different combinations between the two PfGrx(SSG) and three roGFP2 variants from subsequent experiments are shown below. (**c**) Non-reducing SDS-PAGE analysis of intermolecular protein disulfide-bond formation for PfGrx$^{C32S/C88S}$ and PfGrx$^{C88S}$ following treatment with GSSG or Diamide. (**d**) Time-dependent electrophoretic mobility shift assay for the slow dithiol-disulfide exchange between roGFP2$^{WT}$(SH)$_2$ and PfGrx$^{C88S}$(S$_2$). Reduced thiols were modified by mmPEG$_{24}$ before non-reducing SDS-PAGE. Alkylation by mmPEG$_{24}$ adds 1.24 kDa per thiol group. Recombinant roGFP2 and PfGrx variants have a molecular mass of 28.3 and 13.7 kDa, respectively. (**e**) Electrophoretic mobility shift assay according to panel b for roGFP2$^{C151S}$(SH) and PfGrx(S$_2$) did not reveal the formation of a mixed disulfide between both proteins.

**Supplementary Figure 2**

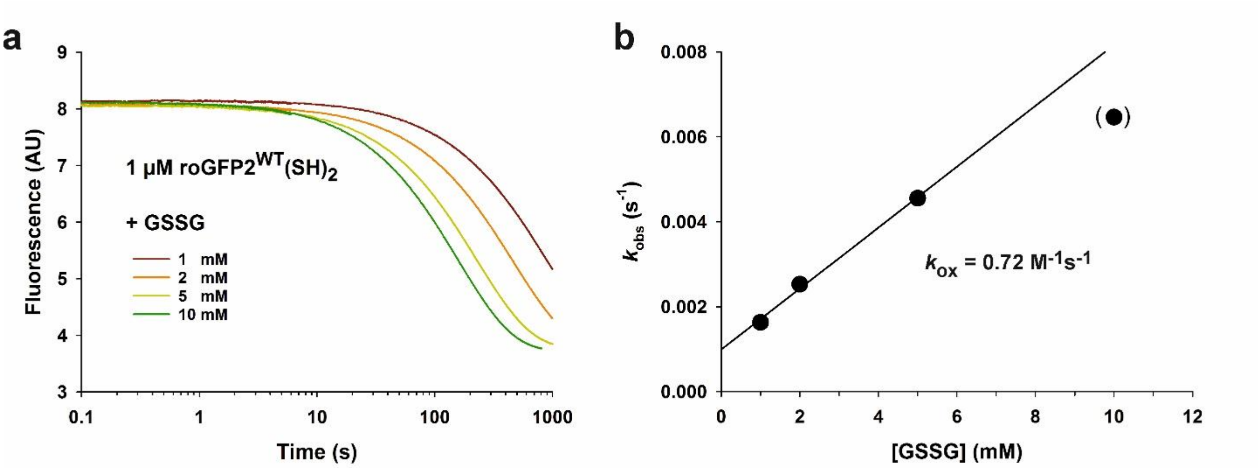


**Supplementary Figure 2 | Nonenzymatic reaction between roGFP2$^{WT}$(SH)$_2$ and GSSG.** (**a**) Representative stopped-flow oxidation kinetics for reduced wild-type roGFP2 at the indicated GSSG concentrations. (**b**) Exponential fits of the data from panel a) yielded $k_{obs}$ values that were plotted against the GSSG concentration. The second order rate constant for the reaction with GSSG was determined from the slope of the linear fit of the secondary plot. The y-axis intercept at $10^{-3}$ $s^{-1}$ might reflect the autoxidation rate of roGFP2$^{WT}$(SH)$_2$.

**Supplementary Figure 3**

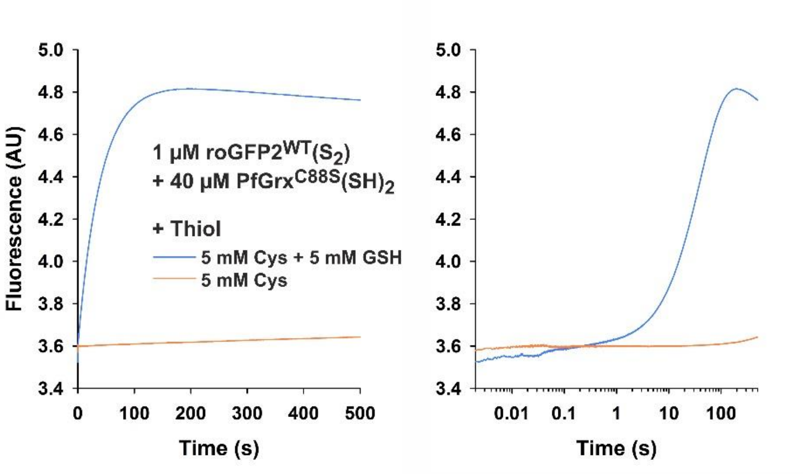


**Supplementary Figure 3 | Thiol-specificity of the PfGrx-catalyzed reduction of roGFP2$^{WT}$(S$_2$).** Representative stopped-flow reduction kinetics for oxidized wild-type roGFP2 with 40 µM reduced PfGrx$^{C88S}$ and 5 mM L-cysteine (Cys) revealed no detectable activity. The subsequent supplementation of the sample with 5 mM GSH served as a positive control. The decrease of fluorescence at the end of the reaction with GSH might reflect a partial reverse reaction with GSSG since no glutathione reductase was added.

**Supplementary Table S1.** *Rate constants at variable GSH concentrations.*

| **Assigned reaction partners A/B/C and products P/Q** | | | | **Rate constant** | **pH/temp.** |
|---|---|---|---|---|---|
| A | + B (+ C) | P | Q | | |
| PfGrx$^{C88S}$(SH)$_2$ | roGFP2$^{WT}$(S$_2$) + 0 mM GSH | PfGrx$^{C88S}$(SH)$_2$ | roGFP2$^{WT}$(SSG) | Not detectable | 8.0/25°C |
| PfGrx$^{C88S}$(SH)$_2$ | roGFP2$^{WT}$(S$_2$) + 1 mM GSH | PfGrx$^{C88S}$(SH)$_2$ | roGFP2$^{WT}$(SSG) | $1.5\times10^{3}$ M$^{-1}$s$^{-1}$ | 8.0/25°C |
| PfGrx$^{C88S}$(SH)$_2$ | roGFP2$^{WT}$(S$_2$) + 2.5 mM GSH | PfGrx$^{C88S}$(SH)$_2$ | roGFP2$^{WT}$(SSG) | $2.1\times10^{3}$ M$^{-1}$s$^{-1}$ | 8.0/25°C |
| PfGrx$^{C88S}$(SH)$_2$ | roGFP2$^{WT}$(S$_2$) + 5 mM GSH | PfGrx$^{C88S}$(SH)$_2$ | roGFP2$^{WT}$(SSG) | $3.4\times10^{3}$ M$^{-1}$s$^{-1}$ | 8.0/25°C |
| PfGrx$^{DM}$(SH) [b] | roGFP2$^{WT}$(S$_2$) + 0 mM GSH | PfGrx$^{DM}$(SH) | roGFP2$^{WT}$(SSG) | Not detectable | 8.0/25°C |
| PfGrx$^{DM}$(SH) [b] | roGFP2$^{WT}$(S$_2$) + 1 mM GSH | PfGrx$^{DM}$(SH) | roGFP2$^{WT}$(SSG) | $2.1\times10^{2}$ M$^{-1}$s$^{-1}$ | 8.0/25°C |
| PfGrx$^{DM}$(SH) [b] | roGFP2$^{WT}$(S$_2$) + 2.5 mM GSH | PfGrx$^{DM}$(SH) | roGFP2$^{WT}$(SSG) | $3.4\times10^{2}$ M$^{-1}$s$^{-1}$ | 8.0/25°C |
| PfGrx$^{DM}$(SH) [b] | roGFP2$^{WT}$(S$_2$) + 5 mM GSH | PfGrx$^{DM}$(SH) | roGFP2$^{WT}$(SSG) | $6.5\times10^{2}$ M$^{-1}$s$^{-1}$ | 8.0/25°C |
| PfGrx$^{C88S}$(SH)$_2$ | roGFP2$^{C151S}$(SSG) + 0 mM GSH | PfGrx$^{C88S}$(SSG) | roGFP2$^{C151S}$(SH) | $4.9\times10^{5}$ M$^{-1}$s$^{-1}$ | 8.0/25°C |
| PfGrx$^{C88S}$(SH)$_2$ | roGFP2$^{C151S}$(SSG) + 1 mM GSH | PfGrx$^{C88S}$(SSG) | roGFP2$^{C151S}$(SH) | $4.2\times10^{5}$ M$^{-1}$s$^{-1}$ | 8.0/25°C |
| PfGrx$^{C88S}$(SH)$_2$ | roGFP2$^{C151S}$(SSG) + 2.5 mM GSH | PfGrx$^{C88S}$(SSG) | roGFP2$^{C151S}$(SH) | $4.0\times10^{5}$ M$^{-1}$s$^{-1}$ | 8.0/25°C |
| PfGrx$^{C88S}$(SH)$_2$ | roGFP2$^{C151S}$(SSG) + 5 mM GSH | PfGrx$^{C88S}$(SSG) | roGFP2$^{C151S}$(SH) | $3.6\times10^{5}$ M$^{-1}$s$^{-1}$ | 8.0/25°C |
| PfGrx$^{C88S}$(SH)$_2$ | roGFP2$^{C208S}$(SSG) + 0 mM GSH | PfGrx$^{C88S}$(SSG) | roGFP2$^{C208S}$(SH) | $1.4\times10^{6}$ M$^{-1}$s$^{-1}$ | 8.0/25°C |
| PfGrx$^{C88S}$(SH)$_2$ | roGFP2$^{C208S}$(SSG) + 1 mM GSH | PfGrx$^{C88S}$(SSG) | roGFP2$^{C208S}$(SH) | $1.1\times10^{6}$ M$^{-1}$s$^{-1}$ | 8.0/25°C |
| PfGrx$^{C88S}$(SH)$_2$ | roGFP2$^{C208S}$(SSG) + 2.5 mM GSH | PfGrx$^{C88S}$(SSG) | roGFP2$^{C208S}$(SH) | $1.1\times10^{6}$ M$^{-1}$s$^{-1}$ | 8.0/25°C |
| PfGrx$^{C88S}$(SH)$_2$ | roGFP2$^{C208S}$(SSG) + 5 mM GSH | PfGrx$^{C88S}$(SSG) | roGFP2$^{C208S}$(SH) | $0.9\times10^{6}$ M$^{-1}$s$^{-1}$ | 8.0/25°C |
| PfGrx$^{DM}$(SH) [a] | roGFP2$^{C151S}$(SSG) + 0 mM GSH | PfGrx$^{DM}$(SSG) | roGFP2$^{C151S}$(SH) | $6.4\times10^{4}$ M$^{-1}$s$^{-1}$ | 8.0/25°C |
| PfGrx$^{DM}$(SH) [a] | roGFP2$^{C151S}$(SSG) + 1 mM GSH | PfGrx$^{DM}$(SSG) | roGFP2$^{C151S}$(SH) | $5.1\times10^{4}$ M$^{-1}$s$^{-1}$ | 8.0/25°C |
| PfGrx$^{DM}$(SH) [a] | roGFP2$^{C151S}$(SSG) + 2.5 mM GSH | PfGrx$^{DM}$(SSG) | roGFP2$^{C151S}$(SH) | $5.2\times10^{4}$ M$^{-1}$s$^{-1}$ | 8.0/25°C |
| PfGrx$^{DM}$(SH) [a] | roGFP2$^{C151S}$(SSG) + 5 mM GSH | PfGrx$^{DM}$(SSG) | roGFP2$^{C151S}$(SH) | $5.5\times10^{4}$ M$^{-1}$s$^{-1}$ | 8.0/25°C |
| PfGrx$^{DM}$(SH) [a] | roGFP2$^{C208S}$(SSG) + 0 mM GSH | PfGrx$^{DM}$(SSG) | roGFP2$^{C208S}$(SH) | $2.5\times10^{5}$ M$^{-1}$s$^{-1}$ | 8.0/25°C |
| PfGrx$^{DM}$(SH) [a] | roGFP2$^{C208S}$(SSG) + 1 mM GSH | PfGrx$^{DM}$(SSG) | roGFP2$^{C208S}$(SH) | $2.7\times10^{5}$ M$^{-1}$s$^{-1}$ | 8.0/25°C |
| PfGrx$^{DM}$(SH) [a] | roGFP2$^{C208S}$(SSG) + 2.5 mM GSH | PfGrx$^{DM}$(SSG) | roGFP2$^{C208S}$(SH) | $2.4\times10^{5}$ M$^{-1}$s$^{-1}$ | 8.0/25°C |
| PfGrx$^{DM}$(SH) [a] | roGFP2$^{C208S}$(SSG) + 5 mM GSH | PfGrx$^{DM}$(SSG) | roGFP2$^{C208S}$(SH) | $2.2\times10^{5}$ M$^{-1}$s$^{-1}$ | 8.0/25°C |

[a] DM = C32S/C88S
