# Supplementary material for: Deciphering the mechanism of glutaredoxin-catalyzed roGFP2 redox sensing reveals a ternary complex with glutathione for protein disulfide reduction
Source: Nat Commun. 2024 Feb 26;15:1733. doi: 10.1038/s41467-024-45808-9 (PMC10897161; doi:10.1038/s41467-024-45808-9)
Supplement: Supplementary file 3 — Description of additional supplementary files [file 41467_2024_45808_MOESM3_ESM.pdf]

Description Of Additional Supplementary Files

Supplementary Data 1: A list of primers and plasmids used to generate samples for this study.
