# Supplementary material for: Deciphering the mechanism of glutaredoxin-catalyzed roGFP2 redox sensing reveals a ternary complex with glutathione for protein disulfide reduction
Source: Nat Commun. 2024 Feb 26;15:1733. doi: 10.1038/s41467-024-45808-9 (PMC10897161; doi:10.1038/s41467-024-45808-9)
Supplement: Supplementary file 5 — Reporting Summary [file 41467_2024_45808_MOESM5_ESM.pdf]

nature portfolio

Corresponding author(s): Marcel Deponte

Last updated by author(s): 25/1/24

Reporting Summary

Nature Portfolio wishes to improve the reproducibility of the work that we publish. This form provides structure for consistency and transparency in reporting. For further information on Nature Portfolio policies, see our Editorial Policies and the Editorial Policy Checklist.

Statistics

For all statistical analyses, confirm that the following items are present in the figure legend, table legend, main text, or Methods section.

n/a	Confirmed	
☐	☒	The exact sample size (*n*) for each experimental group/condition, given as a discrete number and unit of measurement
☐	☒	A statement on whether measurements were taken from distinct samples or whether the same sample was measured repeatedly
☒	☐	The statistical test(s) used AND whether they are one- or two-sided<br>*Only common tests should be described solely by name; describe more complex techniques in the Methods section.*
☐	☒	A description of all covariates tested
☐	☒	A description of any assumptions or corrections, such as tests of normality and adjustment for multiple comparisons
☒	☐	A full description of the statistical parameters including central tendency (e.g. means) or other basic estimates (e.g. regression coefficient) AND variation (e.g. standard deviation) or associated estimates of uncertainty (e.g. confidence intervals)
☒	☐	For null hypothesis testing, the test statistic (e.g. *F*, *t*, *r*) with confidence intervals, effect sizes, degrees of freedom and *P* value noted<br>*Give P values as exact values whenever suitable.*
☐	☒	For Bayesian analysis, information on the choice of priors and Markov chain Monte Carlo settings
☐	☒	For hierarchical and complex designs, identification of the appropriate level for tests and full reporting of outcomes
☒	☐	Estimates of effect sizes (e.g. Cohen's *d*, Pearson's *r*), indicating how they were calculated

Our web collection on statistics for biologists contains articles on many of the points above.

Software and code

Policy information about availability of computer code

Data collection	Pro-Data SX software (Applied Photophysics)
Data analysis	Excel 2016; SigmaPlot 13; kinetic simulations were performed with Pro-KIV 1.01 software (Applied Photophysics)

For manuscripts utilizing custom algorithms or software that are central to the research but not yet described in published literature, software must be made available to editors and reviewers. We strongly encourage code deposition in a community repository (e.g. GitHub). See the Nature Portfolio guidelines for submitting code & software for further information.

Data

Policy information about availability of data

All manuscripts must include a data availability statement. This statement should provide the following information, where applicable:

- Accession codes, unique identifiers, or web links for publicly available datasets
- A description of any restrictions on data availability
- For clinical datasets or third party data, please ensure that the statement adheres to our policy

The authors declare that all data supporting the findings of this study are provided in the Manuscript and its Supplementary Information/Source Data files. The data regarding electrophoretic mobility shift assays, nonenzymatic reaction between roGFP2WT(SH)2 and GSSG, thiol-specificity of the PfGrx-catalyzed reduction of roGFP2WT(S2), and rate constants at variable GSH concentrations are provided in the Supplementary Information. Uncropped and unprocessed scans of Supplementary Figure 1 are provided in the Source Data file. A list of primers and plasmids are provided as separate Supplementary Data.

Research involving human participants, their data, or biological material

Policy information about studies with human participants or human data. See also policy information about sex, gender (identity/presentation), and sexual orientation and race, ethnicity and racism.

Reporting on sex and gender	not applicable
Reporting on race, ethnicity, or other socially relevant groupings	not applicable
Population characteristics	not applicable
Recruitment	not applicable
Ethics oversight	not applicable

Note that full information on the approval of the study protocol must also be provided in the manuscript.

Field-specific reporting

Please select the one below that is the best fit for your research. If you are not sure, read the appropriate sections before making your selection.

☒ Life sciences ☐ Behavioural & social sciences ☐ Ecological, evolutionary & environmental sciences

For a reference copy of the document with all sections, see nature.com/documents/nr-reporting-summary-flat.pdf

Life sciences study design

All studies must disclose on these points even when the disclosure is negative.

Sample size	Data sets were generated from independent biological replicates as shown by individual data points in each figure. No sample size calculation was performed since all measurements from biological replicates were highly reproducible.
Data exclusions	No data were excluded. Only one data point was identified as an outlier (Figure S2B)
Replication	Data sets were generated from at least two independent biological replicates. All attempts at replication were successful.
Randomization	Protein purifications were not randomized but wild-type proteins were studied in parallel for each data series and served as a control for systematic variations (including, for example, new batches of commercial GSH, NADPH etc).
Blinding	Investigators were not blinded. This was not relevant for the kinetic data since all experiments were repeated prior to the final data analysis. The kinetic simulations for each experiment were also not blinded because the calculations are automated.

Reporting for specific materials, systems and methods

We require information from authors about some types of materials, experimental systems and methods used in many studies. Here, indicate whether each material, system or method listed is relevant to your study. If you are not sure if a list item applies to your research, read the appropriate section before selecting a response.

Materials & experimental systems

n/a	Involved in the study	
☒	☐	Antibodies
☒	☐	Eukaryotic cell lines
☒	☐	Palaeontology and archaeology
☒	☐	Animals and other organisms
☒	☐	Clinical data
☒	☐	Dual use research of concern
☒	☐	Plants

Methods

n/a	Involved in the study	
☒	☐	ChIP-seq
☒	☐	Flow cytometry
☒	☐	MRI-based neuroimaging

Plants

Seed stocks	not applicable
Novel plant genotypes	not applicable
Authentication	not applicable
